# Hospital in Hong Kong

**Published:** 1892-04-16

**Authors:** 


### A HOSPITAL IN HONG KONG.

We have receivedthe annual report of the Alice Memorial Hospital, Hong Kong. This institution was founded by Dr. Ho Kai, and handsomely endowed by Mr. Belilios. The house-surgeon is Dr. Chung, the Matron, Mrs. Kwan. In connection with the hospital there is a medical school, under the patronage of His Excellency Li Hung Chang, Viceroy of Chi-Li, in the examinations of which Messrs. Sun Yat Sen, Kong Ying Wa, Wong I Ek, and Wong Kan passed with distinction.

This is not a joke; it is a curious, interesting, and most valuable fact, which shows that in spite of their deep-rooted conservatism, and the fanatical cruelty to which that conservatism has recently given rise, the inhabitants of the Flowery Land are beginning to profit from the knowledge possessed by the "foreign devils" of Europe. It is true that Hong Kong is a British port, and that residents there must perforce submit to the laws of Western Europe, but no laws can make a people accept foreign customs, habits of living, or methods of treating disease. Any such action as this must proceed from a conviction, avowed or not, that the foreign fashion is better than the native. It is true that, much to their own detriment, many patients do not come to the Alice Memorial Hospital until they have exhausted the resources of native medicine; whence it comes that some, though cured, bear about for all their life the visible tokens of their disease, while others are not really cured at all. But it is something gained, among an obstinate people like the Chinese, that they will accept the foreign medicine even as a *dernier ressort.*

As its name indicates, the Alice Hospital is not, in spite of its being founded by a native, a wholly Chinese institution. The visiting surgeons are European, and foreign merchants bulk largely among its supporters. It is, moreover, under the control of the London Missionary Society, and is distinctly not merely a hospital, but a medical mission, with two mission dispensaries affiliated with it. But in face of the success of Mr. Sun Yat Sen and his companions, we may hope that it will some day, while remaining a Christianising institution, be supervised by properly trained native doctors, and tended—is this too much to hope?—by skilled native nurses.

Meanwhile, the record of hospital work throws many interesting lights on Chinese life and opinion. One self-willed damsel, who was being slowly but surely cured of lupus, which had destroyed one eye and eaten up half her face before she came to the Alice Hospital, opined, after six weeks' treatment, that she had given the foreign doctor "plenty good chance," and accordingly went off to consult a native specialist in Canton. Other patients left because they objected to the treatment prescribed, and others because some festival was approaching in whose revelries they wished to share.

Other facts show the superior toughness of the Chinese constitution to the European. It takes a much larger dose of chloroform to anæsthetise the Chinaman; he passes under its influence more slowly, and rarely shows the excitement often characteristic of the initial stage of anæsthesia. Still more rarely does he suffer from sickness on his return to consciousness. This greater apathy of nature helps to recover from severe accidents and operations. For example: On November 28th, 1890, a schoolgirl named Ng A Soo fell from the roof of a four-storeyed house, a distance of sixty or seventy feet. Her skull was fractured in several places, and the right parietal eminence was deeply depressed. Enough to kill anybody, one would say, or at the best to induce lifelong idiocy! Not a bit of it! Dr. Hartigan and Dr. Thomson trephined the skull, taking out a circle five-eighths of an inch in diameter, gouged out a little more, raised the depressed parietal eminence, and in exactly six weeks Ng A Soo left the hospital, "seemingly," says the clinical report, "in no way the worse for her fractured skull." In another case, a patient was able to leave the hospital thirteen days after the removal of an ovarian tumour weighing 64 pounds. They cannot be very sensitive, these Chinese! One man, admitted into the hospital suffering from a large ulcer which extended four inches upward and as much downward from the back of the knee-joint, told how thirty years before he had received a burn there, and the wound, now become an ulcer, had never healed up. Surely he would have another name for the "foreign devils," when, after four months' careful treatment, involving five successive operations, he was at last discharged quite cured.

Among the clinical notes none exceed in interest Dr. Cantlie's observations on leprosy, of which seventy-three cases were treated in the hospital last year. So encouraging are they that we feel justified in quoting them in full:—

"I. The proof of the heredity of leprosy is doubtful; about one-third of the children of lepers become leprous. The children do not become leprous until seven years old, on an average; therefore, they may acquire leprosy by *contact* with their leprous parents.

"II. Lepers seldom have children, and when they do, more than one is the exception.

"III. A healthy child may be born to leprous parents, *both* being lepers; and the child grow up free from leprosy, even though nursed by the leprous mother.

"IV. There are facts to support the statement made by the Chinese that leprosy disappears in the third generation.

"V. Chinese doctors declare they can cure acquired but not hereditary leprosy. The method of cure is by inhalation of mercurial fumes until salivation is induced.

"VI. The spread of leprosy by contagion is slow and irregular. This is well evidenced by the population of the leper settlement at Canton, where there are two hundred lepers and four hundred healthy people, and the inhabitants declare that the accession of lepers to their number is entirely from without."

Dr. Cantlie says that in his experience Unna's ointment, aided by iron and cod liver oil, produce the best results.

The record of ophthalmic practice at the Alice Hospital is full of interest, especially that which tells how the absence of a bridge to his nose causes the Chinaman to be peculiarly liable to entropion. For want of the uplifting power of the nose, the eyelashes turn downward, instead of taking an upward curve, and in such a case a very small irritation will cause disease. To remedy this Dr. Jordan has invented, or combined from Arlt and Dianoux, an operation which, though it somewhat mars the Confucian beauty of the Celestial countenance, gives the eyelashes the upward curve common to mankind in general. Altogether the report of the Alice Hospital, with the training school for Chinese students attached to it, is deeply encouraging, alike from a scientific, religious, and international point of view.

